# Long-term exposure of immortalized keratinocytes to arsenic induces EMT, impairs differentiation in organotypic skin models and mimics aspects of human skin derangements

**DOI:** 10.1007/s00204-017-2034-6

**Published:** 2017-08-03

**Authors:** R. Weinmuellner, K. Kryeziu, B. Zbiral, K. Tav, B. Schoenhacker-Alte, D. Groza, L. Wimmer, M. Schosserer, F. Nagelreiter, S. Rösinger, M. Mildner, E. Tschachler, M. Grusch, J. Grillari, P. Heffeter

**Affiliations:** 1 Christian Doppler Laboratory on Biotechnology of Skin Aging, Department of Biotechnology, BOKU - University of Natural Resources and Life Sciences Vienna, Vienna, Austria; 2 Department of Medicine I, Institute of Cancer Research and Comprehensive Cancer Center Vienna, Medical University of Vienna, Borschkegasse 8a, 1090 Vienna, Austria; 3 Department of Biotechnology, BOKU - University of Natural Resources and Life Sciences Vienna, Muthgasse 18, Haus B, 1190 Vienna, Austria; 4 Department of Dermatology, Medical University of Vienna, Vienna, Austria

**Keywords:** Organotypic culture, Skin equivalents, Arsenic, Immortalized keratinocytes, EMT

## Abstract

Arsenic is one of the most important human carcinogens and environmental pollutants. However, the evaluation of the underlying carcinogenic mechanisms is challenging due to the lack of suitable in vivo and in vitro models, as distinct interspecies differences in arsenic metabolism exist. Thus, it is of high interest to develop new experimental models of arsenic-induced skin tumorigenesis in humans. Consequently, aim of this study was to establish an advanced 3D model for the investigation of arsenic-induced skin derangements, namely skin equivalents, built from immortalized human keratinocytes (NHEK/SVTERT3-5). In contrast to spontaneously immortalized HACAT cells, NHEK/SVTERT3-5 cells more closely resembled the differentiation pattern of primary keratinocytes. With regard to arsenic, our results showed that while our new cell model was widely unaffected by short-time treatment (72 h) with low, non-toxic doses of ATO (0.05–0.25 µM), chronic exposure (6 months) resulted in distinct changes of several cell characteristics. Thus, we observed an increase in the G2 fraction of the cell cycle accompanied by increased nucleus size and uneven tubulin distribution. Moreover, cells showed strong signs of de-differentiation and upregulation of several epithelial-to-mesenchymal transition markers. In line with these effects, chronic contact to arsenic resulted in impaired skin-forming capacities as well as localization of ki67-positive (proliferating) cells at the upper layers of the epidermis; a condition termed Bowen’s disease. Finally, chronically arsenic-exposed cells were characterized by an increased tumorigenicity in SCID mice. Taken together, our study presents a new model system for the investigation of mechanisms underlying the tumor-promoting effects of chronic arsenic exposure.

**Electronic supplementary material:**

The online version of this article (doi:10.1007/s00204-017-2034-6) contains supplementary material, which is available to authorized users.

## Introduction

Arsenic (As) is one of the best known human carcinogens and among the most common environmental pollutants (Sarkar and Paul 2016). Due to its high abundance in the earth crust, there are several regions where high arsenic concentrations in soil and (ground) water represent a significant threat to human health. Thus, it is currently estimated that more than 200 million people are at risk of toxic arsenic exposure (Hunt et al. 2014), of which around 20–45 million people are living in Bangladesh (Hunt et al. 2014; Sen and Biswas 2013; Smith et al. 2000). However, the list of areas at high risk includes besides alluviums of the Indian states also parts of diverse other countries like Thailand, Taiwan, Vietnam as well as the European Union, Canada, the USA and several Latin American regions including Chile, Northern and Central Mexico as well as Brazil (Sarkar and Paul 2016).

In general, intake of arsenic by drinking water is the most important route of chronic intoxication. Besides, irrigation of rice fields with arsenic-contaminated groundwater can also result in chronic arsenic exposure in individuals with nutritional habits heavily based on rice (Gousul Azam et al. 2016; Halder et al. 2012). Overall, inorganic As(III) (e.g., arsenic trioxide, ATO) is the strongest arsenic-based toxin in humans (Sarkar and Paul 2016) and chronic exposure is known to damage a broad range of organ systems in a time- and dose-dependent manner, a condition which is known as arsenicosis (Hunt et al. 2014; Sarkar and Paul 2016; Schuhmacher-Wolz et al. 2009). Thus, individuals suffering from chronic arsenic poisoning experience cardiotoxicity (typically accelerated arteriosclerosis) and enhanced susceptibility to pulmonary infections such as tuberculosis (Hunt et al. 2014; Smith et al. 2011). Most importantly, the WHO list of known human carcinogens places arsenic in group 1 (Pflaum et al. 2016) as it induces (among others) cancers of the lung, skin, bladder, and kidney. Noteworthy, although arsenic affects diverse cellular processes in numerous organs, the symptoms of its toxicity usually first manifest in the skin (Hunt et al. 2014; Sarkar and Paul 2016). These skin abnormalities include hyperpigmentation, hyperkeratosis of the palms and soles, Bowen’s disease, squamous carcinoma and basal cell carcinoma in humans (Hunt et al. 2014). Interestingly, treatment with inorganic arsenic does not induce cancerous skin lesions in most laboratory animals (especially rodents) (Schuhmacher-Wolz et al. 2009; Yajima et al. 2017; Yamanaka et al. 2000, 2001) making the in vivo evaluation of the underlying mechanisms challenging. Thus, it is of high interest to develop experimental models of skin tumorigenesis, which allow the investigation of skin carcinogenesis by arsenicals in humans.

**Fig. 1:**
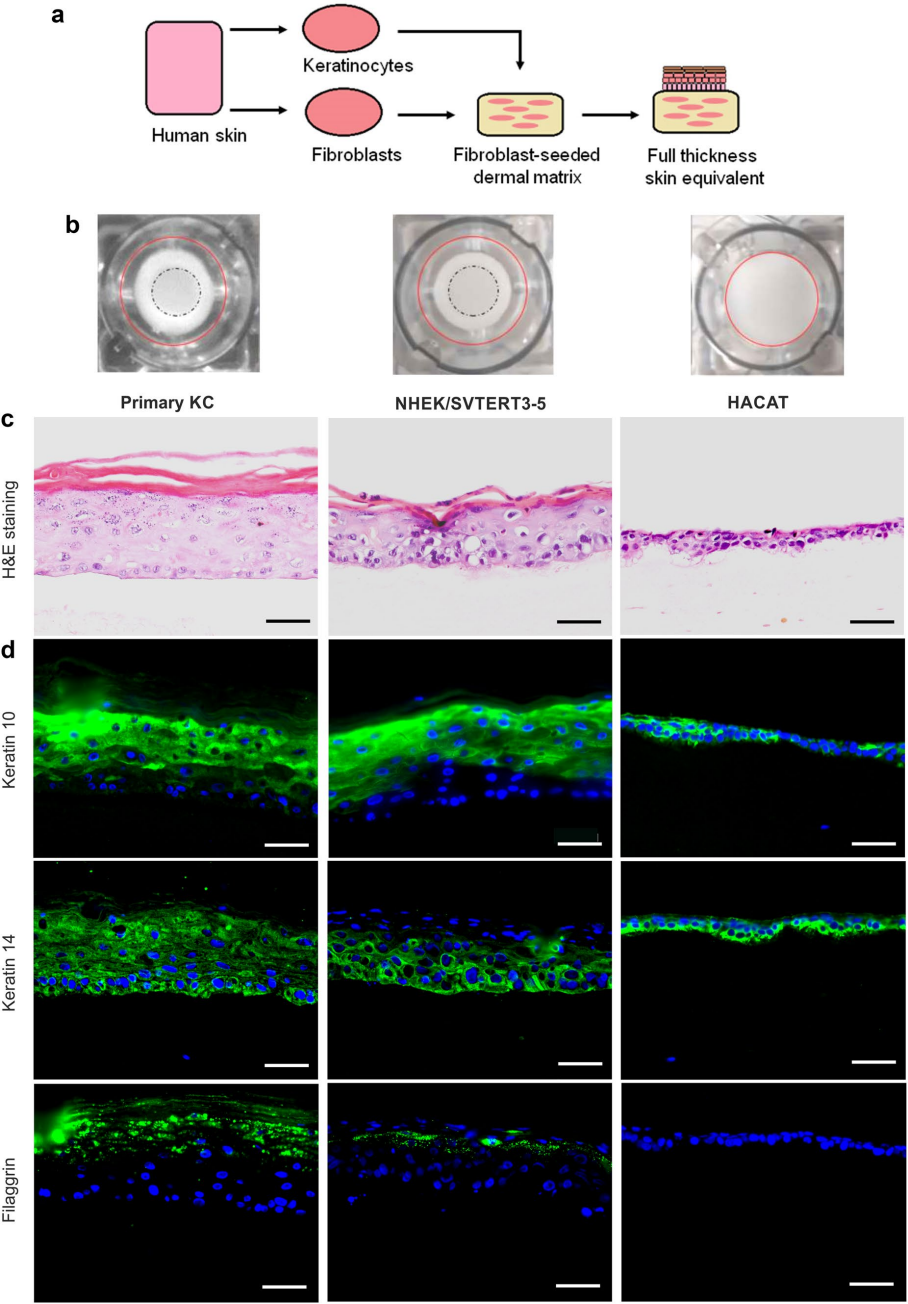
Comparison of human skin equivalents built from primary keratinocytes, NHEK/SVTERT3-5 and HACAT cells. **a** Cartoon is summarizing the preparation of human skin equivalents. **b** Pictures of skin equivalents in transwells before harvesting. The border of the transwell is indicated by a *red*, *solid line*. 3D models built with primary keratinocytes and NHEK/SVTERT3-5 showed contraction of the underlying collagen G matrix with a distinct „ring“ appearance on the outer edges (*dashed lines*), while models built with HACAT cells did not result in visible contraction. **c** H&E staining of human skin equivalents reveals less epidermal layers and *stratum corneum* formation in models built with HACAT compared to samples built from NHEK/SVTERT3-5 cells. **d** Immunohistological evaluation of early (Keratin 10) and late (Filaggrin) differentiation markers as well as the basal layer marker Keratin 14. Pictures are representative of three different experiments. *Scale bar* 50 µm (color figure online)

One option to overcome this problem is the use of organotypic skin equivalents, which are bioengineered substitutes that mimic the human skin (Alepee et al. 2014; Arnette et al. 2016). Today, they are commonly used for two different applications: on the one hand, as skin replacement or graft in clinical routine and, on the other hand, as an alternative to animal models for drug permeability and toxicity testing. In the cosmetic industry, they even have replaced animal testing in regard to skin irritation, erosion and photosensitization. Depending on the complexity of the models, they can be divided into two subgroups: (1) Epidermal or dermal equivalents: three-dimensional models that consist only of keratinocytes (epidermal) or fibroblasts (dermal) growing on a suitable surface, and (2) full-thickness skin equivalents: more advanced models consisting of an artificial fibroblast-containing dermis together with an epidermis built from keratinocytes on top (Arnette et al. 2016; Mathes et al. 2014). Basically, full-thickness skin equivalents are built by preparation of a dermal layer (a collagen matrix populated with fibroblasts) followed by seeding of keratinocytes on top (Fig. 1a). After incubation, the keratinocyte layer is lifted to the air–liquid interface (ALI) and the media composition is changed (Arnette et al. 2016). These stimuli lead to the differentiation of keratinocytes, which subsequently form the typical layered-like structure of human skin over time (*stratum basale*, *stratum spinosum*, *stratum granulosum*, *stratum corneum*) (Matsui and Amagai 2015). Due to their similarity to real human skin, these skin equivalents can be considered as valid test systems for drugs and toxicants, which interact with human skin development and differentiation (Mathes et al. 2014).

Concerning arsenic, there are already reports on skin equivalents build from human adult low calcium high temperature keratinocytes (HACAT) (Klimecki et al. 1997). However, there are several drawbacks when using HACAT cells for these tests, especially as these cells are spontaneously transformed and, thus, lack the expression of several late differentiation markers. Consequently, the aim of this study was to evaluate skin equivalents built from our newly developed NHEK/SVTERT3-5 cells as an advanced 3D model for the investigation of skin disarrangements after chronic arsenic exposure.

## Materials and methods

### Chemicals

Arsenic trioxide (ATO) was purchased from Sigma-Aldrich (MO, USA) and dissolved in 1 M NaOH. For experiments, stocks were further diluted in media to the given concentrations. The final concentration of solvent (NaOH) in all experiments was less than 0.1%. If not indicated otherwise, all reagents used in this study were purchased from Sigma-Aldrich.

### Cell culture

NHEK/SVTERT3-5 were kindly provided by Evercyte, GmbH. Briefly, cells were created by transfecting human keratinocytes isolated from human pendulous abdomen tissue with SV40 early region DNA and subsequently selected for SV40 early region overexpression (NHEK/SV3). In a second step, cells were transduced with retroviral particles containing hTERT as well as a G418 selection marker. The cells were routinely cultured in keratinocyte basal medium 2 (KBM-2) supplemented with KGM-2 SingleQuot© kit (LONZA, Basel, CH) at 37 °C, 7% CO_2_ and 95% humid air. In addition, primary human dermal fibroblasts (HDF, Evercyte, GmbH) were cultured in DMEM/HAMs (1:1, Biochrom, Berlin, GER) supplemented with 10% fetal calf serum (FCS) and 4 mM l-glutamine at 37 °C, 7% CO_2_ and 95% humid air.

Chronically ATO-treated cells were generated by serial passaging of NHEK/SVTERT3-5 cells in cell culture flasks for up to 6 months in the presence of 0 (mock-treated control), 0.05, 0.1 or 0.25 µM ATO added directly to the medium after passaging. All cells were split at a ratio of 1:3 to 1:4 twice a week and periodically tested for absence of mycoplasma. The growth of cells was monitored routinely by cell counting (ViCell X-R, Beckman-Coulter, CA, USA) at each passaging. Population doublings were calculated using the formula *n* = 3.32 × (log (cell count) − log (seeded cells)), whereas *n* is the final population doubling number (Hayflick 1964).

### Cell proliferation assay

Cells were plated (5 × 10^5^/well cells for NHEK/SVTERT3-5 and 3.5 × 10^5^ cells/well for HDF) into 96-well plates and allowed to recover for 24 h. For the investigation of short-term effects, cells were exposed to the indicated concentrations of ATO and incubated for 72 h followed by a resazurin-based vitality assay (AlamarBlue; Thermo Fisher Scientific, MA, USA), which was performed according to manufacturer’s recommendations. Cell viability and IC_50_ values were calculated from full dose–response curves (drug concentrations resulting in 50% reduction of viable cells compared to untreated control cells cultured in parallel) using GraphPad Prism software (La Jolla, USA). If not stated otherwise in the figure legend, data are given as mean from three independent experiments with at least six data points.

### Cell cycle analysis

To investigate short-term effects of ATO treatment, NHEK/SVTERT 3-5 were seeded in 75 cm^2^ flasks. After 24 h of recovery, cells were exposed to the indicated amount of ATO for 48 h. For chronic effects of arsenic treatment, long-term ATO-treated cells were seeded into 75 cm^2^ flasks with their corresponding concentration of ATO. Cells were treated with 10 µM BrdU (dissolved in nuclease-free water) for 24 h. Then, cells were harvested and 300,000 cells were fixed with ice-cold 70% ethanol. Cells were stored overnight at 4 °C, then permeabilized with 2 M HCl/1% Triton X-100 for 30 min. Afterwards, the cell suspension was neutralized with 0.1 M Na-borate (pH 8.5), stained for 30 min with monoclonal mouse anti-BrdU antibody (Becton Dickinson, CA, USA) and counterstained with an anti-mouse FITC-conjugated antibody (1:100, Sigma-Aldrich). For analysis of cell cycle distribution, cells were counterstained with 2.5 µg propidium iodide (PI)/ml and analyzed by flow cytometry using a Gallios Flow Cytometer (Beckman Coulter). Quantification of DNA histograms was done using KALUZA software (Becton Dickinson, CA, USA).

### H_2_DCF-DA-stain

Cells were treated for 1 h with respective concentrations of ATO, harvested, resuspended in phosphate-buffered saline (PBS) containing 10 μM 2′,7′-dichlorodihydrofluorescein diacetate (H_2_DCF-DA; Life Technologies) and incubated for 30 min at room temperature in the dark. After incubation, cells were kept on ice and fluorescence was measured on a Gallios Flow Cytometer (Beckman Coulter). Analysis of data was done using KALUZA software (Becton Dickinson, CA, USA).

### Apoptosis analysis

Chronically ATO-treated cells were seeded in 25 cm^2^ flasks. After 72 h recovery under the respective concentrations of ATO, cells were harvested and centrifuged at 200×*g* for 10 min. Pellets were washed twice with Annexin V-binding buffer (10 mM Hepes/NaOH pH 7.4, 140 mM NaCl, 5 mM CaCl_2_). The pellet was resuspended in Annexin V/PI staining solution (Pacific Blue™ Annexin-V Kit, Biolegend, CA, USA containing 250 ng/ml PI) and incubated for 15 min. Apoptotic cells were analyzed using a Gallios Flow Cytometer (Beckman Coulter) with KALUZA software (Becton Dickinson, CA, USA). As a positive control, staurosporine (40 nM) treatment for 24 h was used.

### Human skin equivalents

The HSE were produced as published by (Mildner et al. 2006) (Fig. 1a). Briefly, 2.5 × 10^5^ HDF were seeded in a collagen gel consisting of eight parts collagen G (Biochrome, Berlin, DE), one part 10 × HBSS (ThermoFisher Scientific, MA, USA) and one part FCS (Sigma-Aldrich, MO, USA). The gel was equilibrated overnight with KGM-2 supplemented with KGM-2 Bullet Kit (Lonza, Basel, CH) followed by a keratinocyte overlay of 1.5 × 10^6^ cells on day 2. The so-formed early skin equivalents were then lifted to the air-liquid interface to start differentiation on day 3. The differentiation media (KGM, Lonza, Basel, CH) was supplemented with all components of the KGM BulletKit (Lonza, Basel, CH) except for bovine pituitary extract. Additionally, 1.15 mM CaCl_2_, 50 µg/ml l-ascorbic acid, 0.1% bovine serum albumin and 10 µg/ml transferrin (all from Sigma-Aldrich) were added. The differentiation medium was refreshed every other day throughout the whole differentiation process (day 3–10). After 10 days, the skin equivalents were harvested, formalin-fixed, and paraffin-embedded for further histological analysis. Additionally, RNA and protein samples were used to confirm the results of the histological analysis. All skin equivalent experiments were performed in duplicates and were repeated three times.

### Immunohistochemistry

All stainings were performed on 5 µm thick sections of formalin-fixed, paraffin-embedded tissues. A standard hematoxylin and eosin (H&E) staining technique was used for histological analyses. Immunohistochemical analysis was performed according to previously established protocols (Filipits et al. 2007). Briefly, after paraffin removal, sample rehydration and blockage of endogenous peroxidase with 0.3% hydrogen peroxide, antigen retrieval with 10 mM citrate buffer (containing 0.05% Tween 20, pH 6.0) was performed for 30 min at 80 °C. The sections were then incubated with the first antibody (Supplementary Table 1) in PBS/2% BSA overnight at 4 °C. After washing with PBS, slides were incubated with the corresponding secondary antibodies in PBS/2% BSA for 1 h at room temperature. The slides for fluorescence analysis were counterstained with DAPI (1:5000, ThermoFisher Scientific, MA, USA), mounted with Fluoprep (bioMérieux, Marcy l’Etoile, France) and analyzed by fluorescence microscopy on a Leica DMI-6000 microscope. Alternatively, visualization was done by treating slides with HRP-conjugated secondary antibodies with ultravision-labeled horseradish peroxidase (HRP) polymer (UVLP, Dako, Glostrup, Denmark) for 15 min. Then, antibody binding was visualized with DAB+ chromogen and counterstained with hematoxylin.

### Immunofluorescence

Cells were seeded into 8-well slides (IBIDI, Martinsried, Germany) and cultivated for 48 h with the indicated concentrations of ATO. After 48 h, cells were washed once with PBS, fixed for 30 min with 4% paraformaldehyde, blocked and permeabilized for 20 min at room temperature with PBS containing 5% FCS and 0.2% Triton X. The cells were then incubated with the first antibody diluted in blocking solution (Supplementary Table 1) overnight at 4 °C. After washing with PBS, cells were incubated with the respective secondary antibody in blocking solution for 1 h at room temperature. For visualization, cells were counterstained with DAPI (1:5000, ThermoFisher Scientific, MA, USA) and analyzed by fluorescence microscopy on a Leica DMI-6000 microscope.

### Spheroid assay

Chronically ATO-treated cells, as well as control cells, were seeded in a low adhesion plate (1000 cells/well) and cultured for 11 days. Half of media was changed every other day and contained the respective concentrations of ATO. Cell growth and viability were evaluated with a resazurin-based vitality assay (AlamarBlue; ThermoFisher Scientific, MA, USA) which was performed according to the manufacturer’s recommendations.

### Western blot analysis

Total protein lysates of membrane-enriched extracts were prepared, separated by SDS-PAGE, and transferred onto a polyvinylidene difluoride membrane for western blotting as described previously (Heffeter et al. 2007). Primary antibodies used are given in Supplementary Table 2. Secondary, horseradish peroxidase-labeled antibodies from Santa Cruz Biotechnology were used in working dilutions of 1:10,000. Since reference proteins were affected by chronic arsenic treatment and could not be used for a reliable normalization, total protein load via Coomassie staining as outlined by (Eaton et al. 2013) was used instead.

### Xenograft experiments

Six- to 8-week-old female CB-17^scid/scid^ mice were purchased from Harlan Laboratories (IN, USA). The animals were kept in a pathogen-free environment and every procedure was done in a laminar airflow cabinet. The experiments were carried out according to the regulations of FELASA and the Ethics Committee for the Care and Use of Laboratory Animals at the Medical University of Vienna (Vienna, Austria). For tumorigenicity tests, 1.5 × 10^6^ cells (in serum-free cell culture medium containing 25% matrigel) were injected subcutaneously into the right flank of the CB-17^scid/scid^ mice. Animals were controlled for distress development every day and caliper measurements regularly assessed tumor size. A positive tumor growth was counted if tumors were palpable and further reached volumes >50 mm^3^ calculated by the formula: (length × width^2^)/2.

### Time-lapse microscopy

2.5 × 10^4^ cells per well were seeded in an 8 well µ-slide (ibidi, Martinsried, Germany), and treated with respective concentrations of ATO. Filming started immediately after treatment (a humidified incubation chamber ensured stable cell culture conditions throughout the experiment (37 °C, 5% CO_2_)). Photomicrographs (20× magnification) were taken with an NIKON Ti fully automatic inverted microscope from Visitron Systems (Puchheim, Germany) every 5 min for 45 h. Cell tracking was performed with the ImageJ/Fiji plugin TrackMate (version 3.4.2.) using an automated approach. Mean cell velocity (µm/min) of >100 cells per group was calculated.

### Statistics

**Fig. 2:**
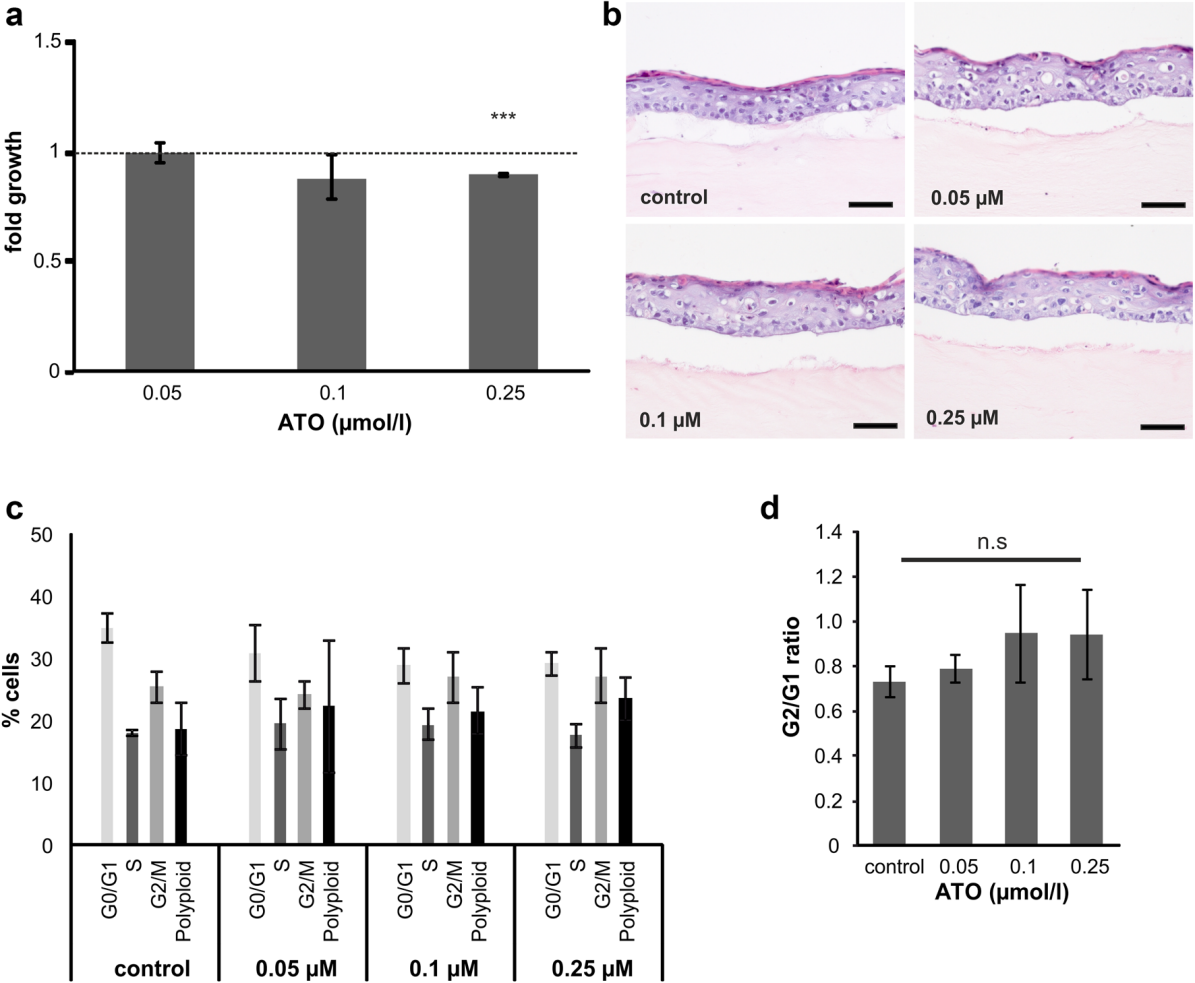
No distinct impact of short-term ATO treatment on immortalized human keratinocytes. **a** NHEK/SVTERT3-5 cells were treated for 72 h with indicated concentrations of arsenic trioxide (ATO) or solvent. After incubation, cell growth was measured by AlamarBlue viability assay. Only cells treated with the highest dose (0.25 µM) show slightly but significantly reduced growth (*p*<0.01 by ANOVA test). Data represent the mean from three independent experiments containing six replicates. Solvent-treated control cells are set to 1 and are represented as *dotted line*. **b** H&E staining of human skin equivalents built with immortalized keratinocytes, which were treated for 7 days with indicated concentrations of ATO followed by formalin-fixation and paraffin embedding. Pictures are representative of three independently performed experiments. *Scale bar* 50 µm. **c** Changes in the cell cycle distribution of NHEK/SVTERT3-5 after treatment for 72 h with the indicated concentrations of ATO were analyzed by PI staining and flow cytometry. Percentages of cells in G0/G1, S and G2/M phase are indicated. **d** Calculated G2/G1 ratio of the obtained data

All measurements were performed in triplicates in three independent experiments if not stated otherwise in the figure legend. Data are expressed as mean ± SD. For comparison between mock-treated (control) and ATO-treated cells, one-way ANOVA following Tukey’s test was used except for Fig. 2c and Supplementary Fig. 2a where a *z* test following Yates correction was used. For the animal experiment, two-way ANOVA analyses with “treatment” and “days” as calculation parameters were performed using GraphPad Prism software. *p* values below 0.05 were considered as statistically significant and marked with stars: *p<0.05; **p<0.01; ***p<0.001.

## Results

### Skin equivalents (HSE) from NHEK/SVTERT 3-5 cells resemble the morphology of primary keratinocytes in 3D culture

To establish a convenient model system, we compared full-thickness skin equivalents formed (1) by primary keratinocytes, (2) by the immortalized keratinocyte cell line (NHEK/SVTERT3-5; generated by overexpression of SV40 early region and the catalytic subunit of human telomerase), and (3) samples built from the tumor cell line HACAT. While skin equivalents from NHEK/SVTERT3-5 were similar in morphology and differentiation pattern to primary keratinocytes, HSEs of HACAT were not able to contract the underlying collagen dermis resulting in a macroscopically visible larger diameter of the samples (Fig. 1b). Subsequent histological evaluation (Fig. 1c) confirmed that HSEs from HACAT cells were overall thinner with fewer cell layers than the ones derived from NHEK/SVTERT 3-5 and primary keratinocytes. Moreover, NHEK/SVTERT3-5 HSE were (in contrast to HACAT) characterized by a more physiological keratin pattern, with keratin 14 (proliferation marker) distributed in the lower layers, keratin 10 (early differentiation marker) in the upper layers and filaggrin (late differentiation marker) in the stratum granulosum of the HSEs (Fig. 1d).

### No distinct impact of short-term ATO treatment on NHEK/SVTERT3-5

To test short-term effects of arsenic, NHEK/SVTERT3-5 as well as human dermal fibroblasts (HDF) were exposed for 72 h to arsenic trioxide (ATO). IC_50_ values were 6.8 and 29.6 µM for keratinocytes and fibroblasts, respectively (Supplementary Fig. 1a and b). In general, the concentrations of arsenic in drinking water varies between the regions at risk [e.g., in some parts of Taiwan, Argentina, the US or China concentrations >3000 µg/l are found (Hunt et al. 2014)]. As WHO recommends arsenic concentrations in drinking water to be lower than 10 µg/l (0.13 µM) (Liang et al. 2017), we selected ATO concentrations of 0.05 µM, 0.1 µM and 0.25 µM for all subsequent experiments.

Out of these concentrations, only 0.25 µM caused minor effects on cell proliferation of our immortalized human keratinocytes resulting in ~10% decrease in final cell number after 72 h of treatment (Fig. 2a). Additionally, skin equivalents constructed using short-term ATO-treated keratinocytes did not show any changes in epidermal thickness nor composition of the epidermal layers when histologically evaluated (Fig. 2b). An analysis of the cell cycle distribution in 2D conditions revealed no significant changes in the G2/G1 ratio (Fig. 2c), although a trend towards an increased percentage of cells in the G2/M phase was observed in samples treated with 0.1 or 0.25 µM ATO (Fig. 2d). Together, this indicates that short-term treatment with environmentally relevant arsenic concentrations had no distinct impact on the analyzed parameters in NHEK/SVTERT3-5.

### Chronic exposure with ATO leads to enhanced arsenic sensitivity and an accumulation of cells in the G2 phase of the cell cycle

**Fig. 3:**
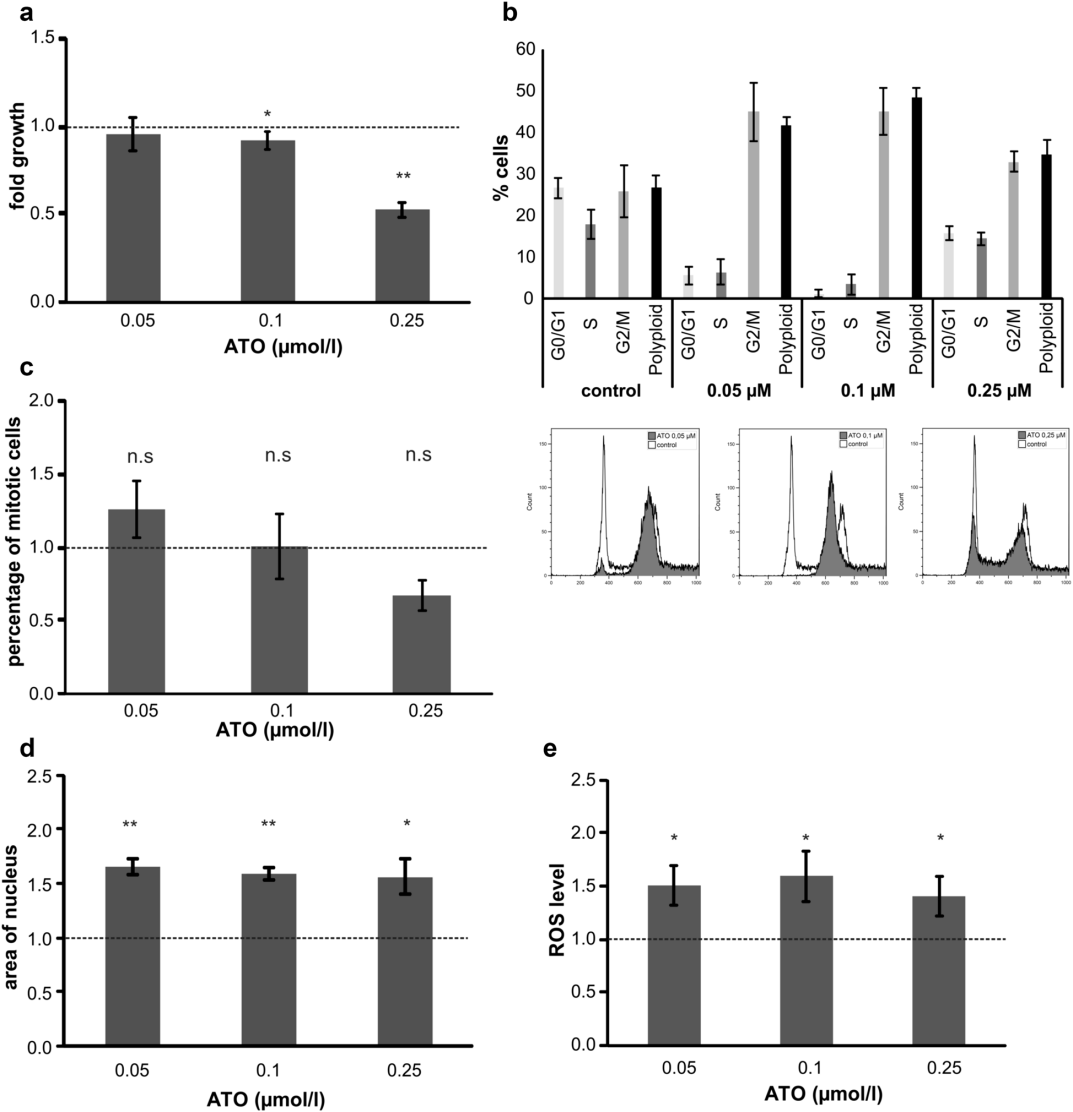
Chronic exposure with ATO leads to enhanced arsenic sensitivity and an accumulation of cells in the G2 phase of the cell cycle. NHEK/SVTERT3-5 were treated for 6 months with indicated concentrations of arsenic trioxide (ATO) or solvent before experiments were performed. **a** Sensitivity to ATO was assessed by AlamarBlue viability assay after 72 h treatment. Values are given relatively to the untreated controls set as 1 (*dotted line*) and represent means and SD of three experiments containing each six replicates. **b** The impact of short-term ATO treatment (72 h) on the cell cycle distribution of NHEK/SVTERT3-5 chronically exposed to the indicated concentrations of ATO was analyzed by PI staining and flow cytometry. Cells were treated with the ATO concentration corresponding to their selection pressure. Percentages of cells in G0/G1, S and G2/M phase are indicated. **c** The impact of ATO treatment on chronically ATO-exposed NHEK/SVTERT3-5 cells at the indicated concentrations was investigated by DAPI stain of acetone/ethanol-fixed cells. Percentage of cells with mitotic features was determined after the indicated ATO treatment (corresponding to their chronic selection pressure) for 72 h. In total, 300–500 nuclei of at least two slides for each concentration were analyzed. Solvent-treated control cells are set to 1 and are represented as *dotted line*. **d** Expansion of nuclear size after chronic exposure to ATO was measured by ImageJ in pictures of DAPI-stained samples. Solvent-treated control cells are set to 1 and are represented as *dotted line*. **e** To assess ROS levels resulting from ATO treatment, chronically ATO-exposed cells were treated for 1 h with the indicated ATO dose (corresponding to their chronic selection pressure) and examined by H_2_DCF-DA-stain. Values are given relatively to the untreated controls set as 1 (*dotted line*) and represent means and SD of two experiments in triplicates

To study long-term arsenic effects on immortalized keratinocytes, we cultivated them in the presence of ATO (0.05, 0.1, 0.25, and 0.5 µM) and monitored their growth. Noteworthy, treatment with 0.5 µM ATO was toxic to the cells resulting in loss of the cell line after the first passage. In contrast, concentrations <0.5 µM had no significant impact on the population doubling time of the cells (Supplementary Fig. 1c). After 6 months of treatment, the resulting cell lines were analyzed for cell proliferation and cell cycle distribution. Interestingly, despite their long exposure to ATO, the resulting cell lines were even more sensitive to treatment than the chemo-naive cells (Fig. 3a, compare to Fig. 2a), as treatment with 0.25 µM resulted in a 47% reduction in final cell number within 72 h. Additionally, cells treated with 0.1 µM ATO showed a significant reduction in cell proliferation (~10%; Fig. 3a). Analysis of the cell cycle distribution using PI staining indicated a marked increase of cells in G2/M phase as well as an increase in the fraction of polyploid cells upon ATO treatment (Fig. 3b). However, subsequent analysis of the nuclear morphology by cytospin and DAPI staining (Fig. 3c) revealed no statistically significant decrease of mitotic cells in all arsenic-treated cell lines, indicating that the increase of the G2/M fraction seen in the PI staining might be due to an enhanced G2 population. Additionally, no significant increase in apoptosis was seen (Supplementary Fig. 2a). Interestingly, we observed that cells under chronic ATO treatment were characterized by a significantly larger nucleus (~1.5-fold increase in nucleus area) and an uneven tubulin distribution (Fig. 3d and Supplementary Fig. 2b) as compared to untreated controls, which could together indicate a higher fraction of aberrant mitosis.

Since high levels of ATO lead to generation of ROS in diverse cell types including HACAT, mouse skin fibroblasts and hepatocytes (Chayapong et al. 2016; Choudhury et al. 2016; Mir et al. 2017), we also tested our new models for occurrence of ROS upon arsenic treatment. Indeed, even treatment with the lowest concentration of 0.05 µM ATO resulted in significantly increased ROS levels (Fig. 3e) shortly after incubation. Overall, these experiments indicate that despite diverse cellular changes, chronic exposure to non-toxic levels of arsenic treatment does not result in activation of ATO resistance mechanisms in NHEK/SVTERT3-5 cells.

### Chronic ATO treatment results in loss of cell differentiation capability in immortalized human keratinocytes in 2D and 3D conditions

**Fig. 4:**
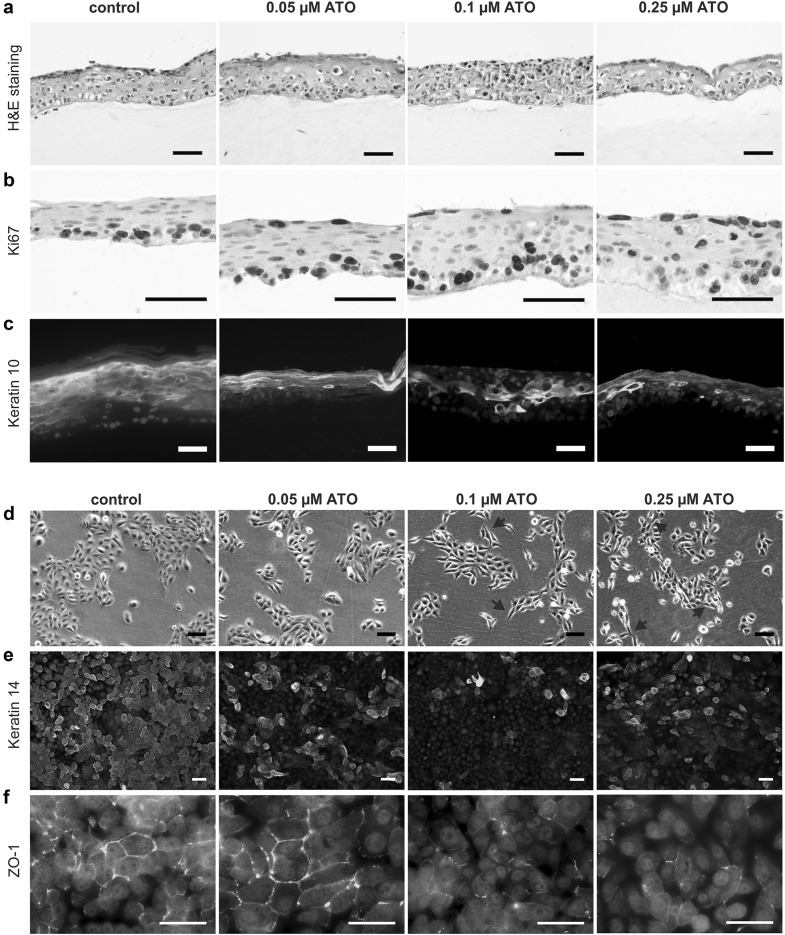
Chronic ATO treatment results in loss of cell differentiation capability in immortalized human keratinocytes in 2D and 3D conditions. **a** Skin equivalents were built from cells chronically treated with the indicated concentrations of ATO. After 7 days at air-liquid interface, samples were formalin-fixed, paraffin-embedded and stained **a** with H&E, **b** for the proliferation marker Ki67, and **c** the early differentiation marker keratin 10. All pictures shown are representative of experiments performed in triplicates. *Scale bar* 50 µm. **d** Bright field microscopic pictures of cells chronically treated with the indicated concentrations of ATO. Arrows point to elongated cells that lost their epithelial appearance. 2D cell layers of chronically ATO-exposed cells were formalin-fixed and immunohistologically stained for the basal cell marker **e** keratin 14 and **f** the tight-junction marker ZO-1. *Scale bar* 20 µm

To test whether long-term ATO-exposed keratinocytes are still able to differentiate, we used our chronically ATO-exposed cells (in comparison to mock-treated samples) for the generation of HSEs. As shown in Fig. 4a, chronic contact to arsenic resulted in impaired skin-forming capacities. Especially the equivalents built after treatment with 0.1 µM ATO showed a distinct loss of differentiation layers and stratification. Thus, in ATO concentrations higher than 0.05 µM proliferating cells were not only confined to the *stratum basale* (which would be expected for healthy tissue), but could be localized even in the *stratum corneum*, which was also confirmed by Ki67 stain (Fig. 4b). Moreover, the loss of differentiation capacity was proven by immunohistochemical staining for the early differentiation marker keratin 10 (Fig. 4c), where chronically ATO-treated keratinocytes exhibited an aberrant differentiation pattern with keratin 10 loss in the upper layers of the epidermis.

**Fig. 5:**
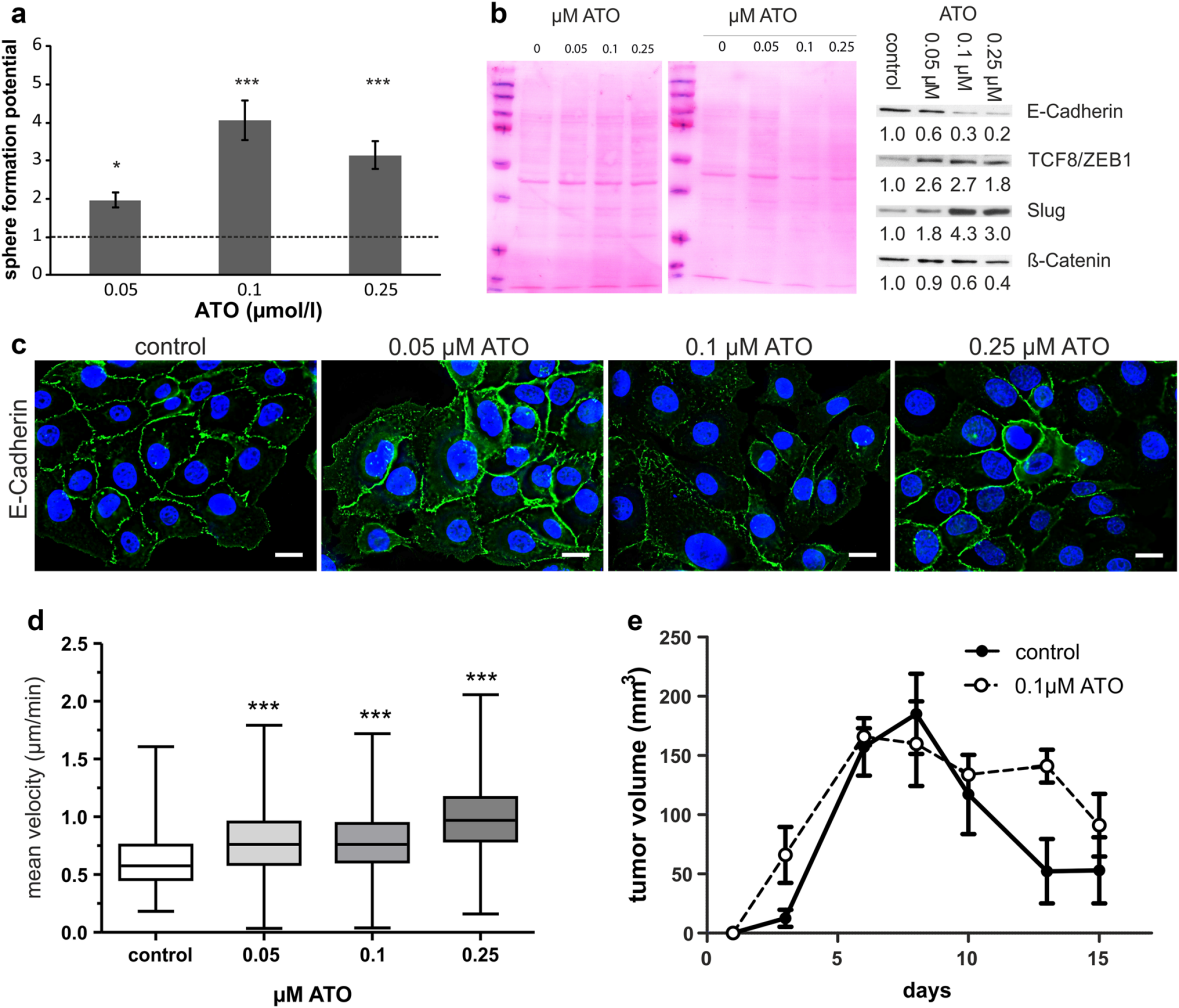
Chronic, low-level ATO exposure leads to EMT and enhanced invasiveness of immortalized human keratinocytes. **a** Evaluation of sphere formation capability was done in low adhesion plates. The growth of cells was measured by Alamar Blue assay. Values are given relatively to the untreated controls set as 1 (*dotted line*) and represent means and SD of two experiments in triplicates. **b** Expression levels of diverse proteins involved in EMT determined in chronically ATO-treated cells after treatment with ATO for 24 h. Band intensity was quantified using ImageJ and normalized to the respective Ponceau staining (*left*). **c** 2D cell layers of chronically ATO-exposed cells were formalin-fixed and immunohistologically stained for E-cadherin. Nuclei were counterstained blue with DAPI. *Scale bar* 10 µm. **d** Chronically treated NHEK/SVTERT3-5 cells were seeded into µ-slides (8 well) under exposure to their respective selection pressure of ATO. Cell movement was recorded by taking a photomicrograph (×20 magnification) every 5 min for 45 h. Speed of movement was subsequently analyzed using Fiji/ImageJ TrackMate plugin. **e** Tumorigenicity of the indicated chronically ATO-treated NHEK/SVTERT3-5 cells was evaluated in SCID mice. To this end, 1.5 × 10^6^ cells (in cell culture medium containing 25% matrigel) were injected subcutaneously into the right flank and tumor size was assessed regularly by caliper measurement. Tumor volume was calculated as explained in the “Materials and methods” section

In line with these data, the ATO-exposed keratinocytes exhibited several changes with regard to differentiation and epithelial morphology in 2D culture as well. Besides loss of epithelial shape and switch to a more spindle-like morphology (Fig. 4d), expression of keratin 14, a basal keratinocyte marker, as well as ZO-1, a marker for the formation of tight junctions, was strongly deregulated and reduced (Fig. 4e, f). This suggests that treatment with low-levels of arsenic strongly impacts on the differentiation program of human keratinocytes, which can be considered as a first step towards development of Bowen’s disease and tumor formation, typical hallmarks of arsenicosis (Hunt et al. 2014; Sarkar and Paul 2016; Schuhmacher-Wolz et al. 2009). Accordingly, long-term treatment with ATO also resulted in significantly enhanced sphere-forming capacity (Fig. 5a).

### Chronic, low-level ATO exposure leads to EMT and enhanced invasiveness of immortalized human keratinocytes

Next, we investigated if the changes in cell morphology and differentiation capability could be attributed to an epithelial-to-mesenchymal transition (EMT). One of the first markers of EMT is loss of E-cadherin (Jiang et al. 2013). In line with the loss of keratin-14 and ZO-1, also expression of E-cadherin was considerably lowered for all ATO-treated cell lines as shown by immunofluorescence staining (Fig. 5c) and Western blot (Fig. 5b). Additionally, protein levels of the EMT markers TCF8/ZEB1 and slug were increased (Fig. 5b). Moreover, in migration experiments using transwell chambers, we observed a trend towards an enhanced migration capability for cells treated with higher doses of arsenic (0.1 and 0.25 µM, data not shown). This was also confirmed by time-lapse microscopy (Fig. 5d and Supplementary Fig. 3), where the mean migration speed of all chronically ATO-treated cells was significantly increased (P<0.001 by one-way ANOVA following Tukey’s test). Finally, we tested whether chronic exposure to arsenic resulted in enhanced tumorigenicity in vivo. To this end, NHEK/SVTERT3-5 cells, which underwent chronic treatment with 0.1 µM arsenic or vehicle control, were injected subcutaneously into the right flank of female SCID mice (Fig. 5e). In these experiments, both cell models only resulted in transient tumor formation. However, cells which experienced chronic arsenic exposure, were able to form tumors significantly faster and for a longer time than untreated ones (*p*<0.05 by Two-Way ANOVA) indicating that chronic exposure to low levels of arsenic might indeed represent one step towards enhanced tumorigenicity.

## Discussion

Arsenic is one of the most important human carcinogens, which as a first symptom results in the development of diverse skin derangements. However, the investigation of the mechanisms underlying this malignant deformation of human keratinocytes induced by chronic (low level) arsenic exposure is difficult due to the lack of suitable in vivo and in vitro models. Thus, for example, the development of mouse models is significantly hampered due to interspecies differences in arsenic metabolism (Michailidi et al. 2015) and by the fact that in rodents arsenic is mainly a co-carcinogen (Hunt et al. 2014; Martinez et al. 2011). Consequently, the development of cell culture-based 3D models which strongly resemble the human skin tissue is of high interest. Noteworthy, so-called organotypic cocultures (OCs) or skin equivalents (SE) have been shown to reflect the basic elements of normal human skin development (Klimecki et al. 1997) and were used successfully over the last years to study diverse aspects of skin formation and diseases [e.g., skin aging or the impact of diverse natural products (Ali et al. 2015) and Weinmuellner et al. manuscript in preparation]. One problem associated with conventional cell culture models, however, is that primary keratinocytes have a limited proliferative capacity in vitro and, thus, are not suited for long-term (pollutant or drug) exposure studies.

Hence, we here used an immortalized, primary-like and non-tumorigenic cell line (NHEK/SVTERT3-5) to investigate, if these cells are capable of forming 3D skin equivalents. In contrast to spontaneously immortalized HACAT cells, these skin equivalents were characterized by a multilayered epidermis including a *stratum corneum*. In addition, early as well as late differentiation marker patterns were comparable to those of skin equivalents built with primary keratinocytes, suggesting that this model cell line resembles the behavior of primary cells.

Based on our promising results that NHEK/SVTERT3-5 are reflecting the physiology of primary keratinocytes more closely than e.g., HACAT cells, we hypothesized that this novel system might be of high value for the investigation of arsenic-induced skin derangements. Hence, we tested short-term treatment with low concentrations of arsenic. Overall, the NHEK/SVTERT3-5 cell line-derived data are well in agreement with published data on primary keratinocytes (Germolec et al. 1997; Liao et al. 2011). While NHEK/SVTERT3-5 cells were widely unaffected by short-term treatment with low doses of ATO (0.05-0.25 µM), chronic exposure resulted in a decrease of growth and a general shift in cell cycle distribution towards G2 (Liao et al. 2011). In contrast, HACAT cells have been reported not to respond with reduced viability or changed cell cycle distribution to arsenic treatment up to 0.5 µM (Klimecki et al. 1997), suggesting that they are not sufficiently reflecting the primary keratinocyte biology.

Furthermore, arsenic treatment has repeatedly been reported to compromise the cellular redox system leading to an increase in ROS formation, which correlates with increased DNA amplification, sister chromatid exchange, and chromosome aberrations (Lee et al. 1988). Moreover, chronic low-dose treatment with arsenic led to increased cell survival and impaired apoptosis (Liao et al. 2011). Such an increase in intracellular ROS levels was also observed, when chronically exposing NHEK/SVTERT3-5 to ATO even at the lowest concentration used, while apoptosis levels did not increase. Furthermore, we observed an uneven tubulin distribution especially in the periphery of the nucleus and a 1.5-fold increase of the nuclear area of treated cells. A combination of all these factors may at least partially explain the increase in polyploid cells that we encountered in arsenic-treated cells and might point towards a pro-survival effect on aberrant cells. This further strengthens the hypothesis stated by (Ouyang et al. 2007) that chronic arsenic treatment may promote skin tumor formation by enhanced survival of cells with genetic alterations, which would normally be eliminated by apoptosis.

In addition, while cultivating the cells under the influence of arsenic, we also observed a visible morphological change of the cells from epidermal cobble-stone towards a more elongated, spindle-like phenotype. Since several studies have shown that arsenic-induced malignant transformation is frequently accompanied by cellular morphology changes resembling EMT (Eckstein et al. 2017; Person et al. 2015; Wang et al. 2013), we investigated EMT markers after long-term arsenic exposure. Indeed, we observed loss of keratin 14 and ZO-1 together with an increase in spheroid formation capability in all chronically treated cells. Additionally, TCF8/ZEb1 and Slug (SNAI2) expression increased, while E-cadherin expression decreased, which are again typical signs for EMT. In general, these findings are in line with a study from (Jiang et al. 2013), where treatment of HACAT cells with 1 µM arsenic also led to enhanced spheroid formation and upregulation of Snail 1 (SNAI1), another member of the Snail transcription factor family. Furthermore, functional assays demonstrated (comparable to data on HACAT by (Liu et al. 2015)) an enhanced cellular 2D migration ability of our chronically arsenic-treated NHEK/SVTERT3-5, additionally supporting the idea of arsenic-inducing EMT.

Finally, the possibility of long-term exposure of primary-like keratinocytes in combination with skin equivalents resulted in visualizing arsenic effects comparable to human skin biopsies of patients suffering from chronic arsenic exposure (Hunt et al. 2014). The similarities comprise a clear-cut differentiation deficiency after 6 months of low-level arsenic exposure, an enhanced cell proliferation rate, as well as localization of proliferating cells at the upper layers of the epidermis, a condition well connected to arsenic intoxication and termed Bowen’s disease (skin carcinoma in situ). Later stages of tumorigenesis, such as hyperkeratosis or invasion into the collagen matrix, were not observed in our model system, in contrast to reports on HACAT cells (Klimecki et al. 1997). This might reflect the fact that HACAT cells are per se closer to tumorigenesis than NHEK/SVTERT3-5 cells, even though the latter carry 2 out of 3 hits that are considered to be sufficient for full tumorigenic transformation of normal cells (Hanahan and Weinberg 2000). Nevertheless, our new cell model shows a significantly enhanced capability to form transient tumors in SCID mice. Interestingly, no stable tumor formation was observed, which might indicate that the chronic exposure to these low-level concentrations of arsenic provides just a further impulse towards enhanced survival and cell growth but might not be sufficient for a full-blown malignant transformation of human keratinocytes. This might support the hypothesis that arsenic needs other supporting factors (UV radiation or mechanically induced repair stress) for execution of its tumorigenic potential.

Taken together, present model system based on NHEK/SVTERT3-5 cells is a suitable tool to investigate the molecular mechanisms underlying arsenicosis as a pre-requisite for identifying treatment options. In addition, it might be fit for purpose in testing effects of chronic pollutant or drug exposure on the human skin.

## Electronic supplementary material

Below is the link to the electronic supplementary material.

Impact on arsenic on cell growth. (a) Human dermal fibroblasts (HDF) and chemo-naive NHEK/SVTERT3-5 cells (b) were treated with indicated concentrations of arsenic trioxide (ATO) or solvent. After 72 h incubation, cell viability was measured by AlamarBlue viability assay. Data represent the mean from three independent experiments containing six replicates. IC50 values were 29.6 µM and 6.8 µM, respectively. (c) Effects of chronic arsenic treatment on cell growth is shown. Cells were split twice a week and cell growth was measured by AlamarBlue viability assay. Population doublings were calculated as explained in the material and methods section and are given on the y-axis. The x-axis reflects the days under chronic ATO treatment. (TIFF 49972 kb)

Chronic arsenic treatment has no effect on apoptosis levels in NHEK/SVTERT3-5 cells. (a) The impact of ATO treatment on chronically ATO-exposed NHEK/SVTERT3-5 cells at the indicated concentrations was investigated by AnnexinV/PI staining and subsequent FACS analysis. Percentage of cells with apoptotic features was determined after the indicated ATO treatment (corresponding to their chronic selection pressure) for 72 h. (b) 2D cell layers of chronically ATO-exposed cells were formalin-fixed and immunhistologically stained with tubulin tracker. Scale bar, 50 µM (TIFF 38047 kb)

Representative example of single cell migration trajectories. Migration trajectories were generated with Fiji/ImageJ using the TrackMate plug-in and Simple LAP tracker from time-lapse microscopy images. (TIFF 18701 kb)

Supplementary material 4 (DOCX 12 kb)

Supplementary material 5 (DOCX 12 kb)

## Abbreviations

ALI: Air-liquid interface
ANOVA: Analysis of variance
ATO: Arsenic trioxide
BCRP: Breast cancer resistance protein
BSA: Bovine serum albumin
DMSO: Dimethyl sulfoxide
EMT: Epithelial-to-mesenchymal transition
H_2_DCF-DA: 2′,7′-Dichlorodihydrofluorescein diacetate
FCS: Fetal calf serum
HACAT: Human adult low Calcium high Temperature keratinocytes
HBSS: Hanks buffered salt solution
HDF: Human dermal fibroblasts
H&E: Hematoxylin and eosin
HRP: Horseradish peroxidase
HSE: Human skin equivalents
OC: Organotypic (co)cultures
PBS: Phosphate-buffered saline
PI: Propidium iodide
ROS: Reactive oxygen species
SE: Skin equivalents
