# Supplementary figures and images for: Long-term exposure of immortalized keratinocytes to arsenic induces EMT, impairs differentiation in organotypic skin models and mimics aspects of human skin derangements
Source: Arch Toxicol. 2017 Aug 3;92(1):181–94. doi: 10.1007/s00204-017-2034-6 (PMC5773649; doi:10.1007/s00204-017-2034-6)

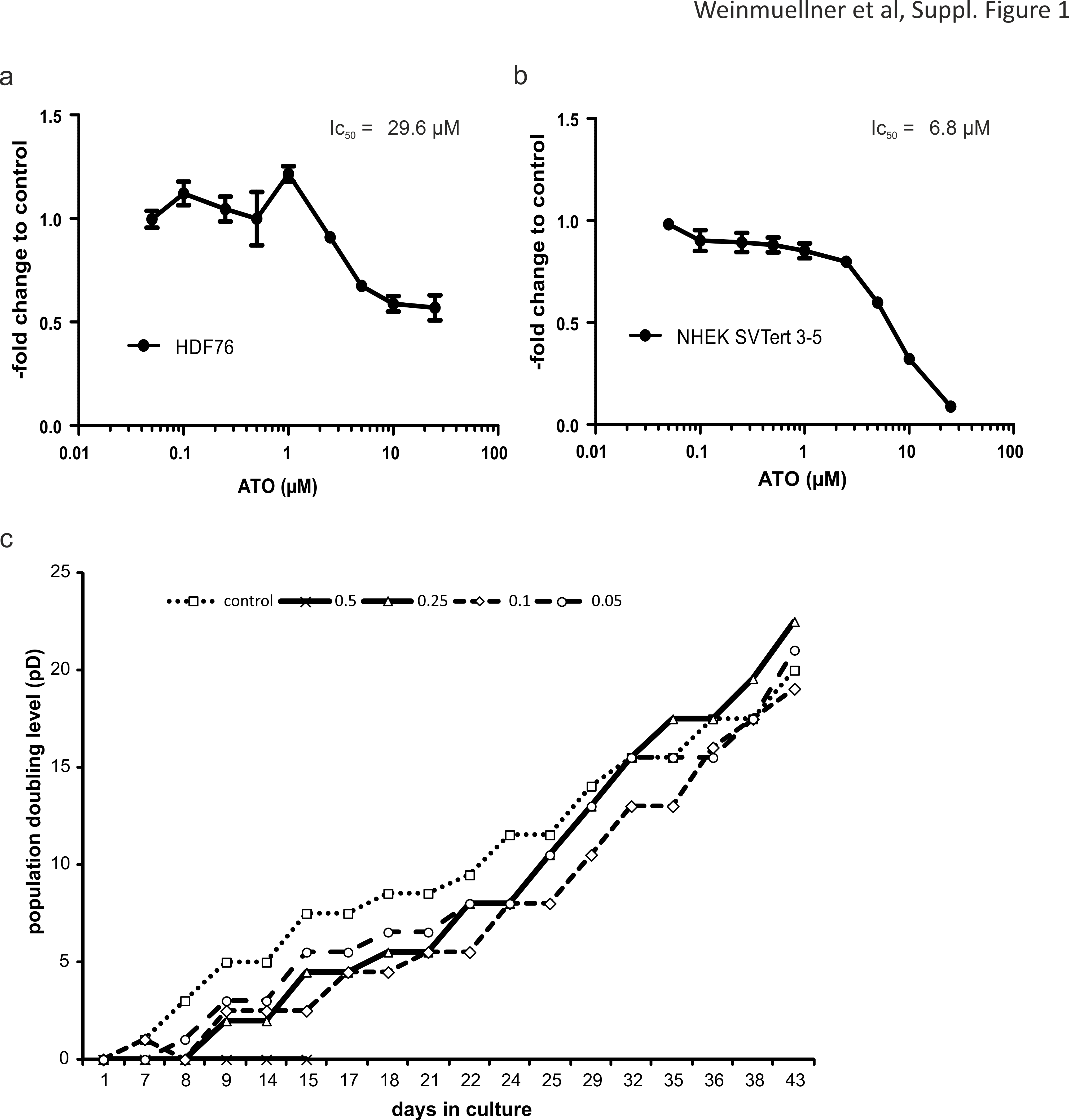

Supplement: Supplementary file 1 — Impact on arsenic on cell growth. (a) Human dermal fibroblasts (HDF) and chemo-naive NHEK/SVTERT3-5 cells (b) were treated with indicated concentrations of arsenic trioxide (ATO) or solvent. After 72 h incubation, cell viability was measured by AlamarBlue viability assay. Data represent the mean from three independent experiments containing six replicates. IC50 values were 29.6 µM and 6.8 µM, respectively. (c) Effects of chronic arsenic treatment on cell growth is shown. Cells were split twice a week and cell growth was measured by AlamarBlue viability assay. Population doublings were calculated as explained in the material and methods section and are given on the y-axis. The x-axis reflects the days under chronic ATO treatment. (TIFF 49972 kb) [file 204_2017_2034_MOESM1_ESM.tif]

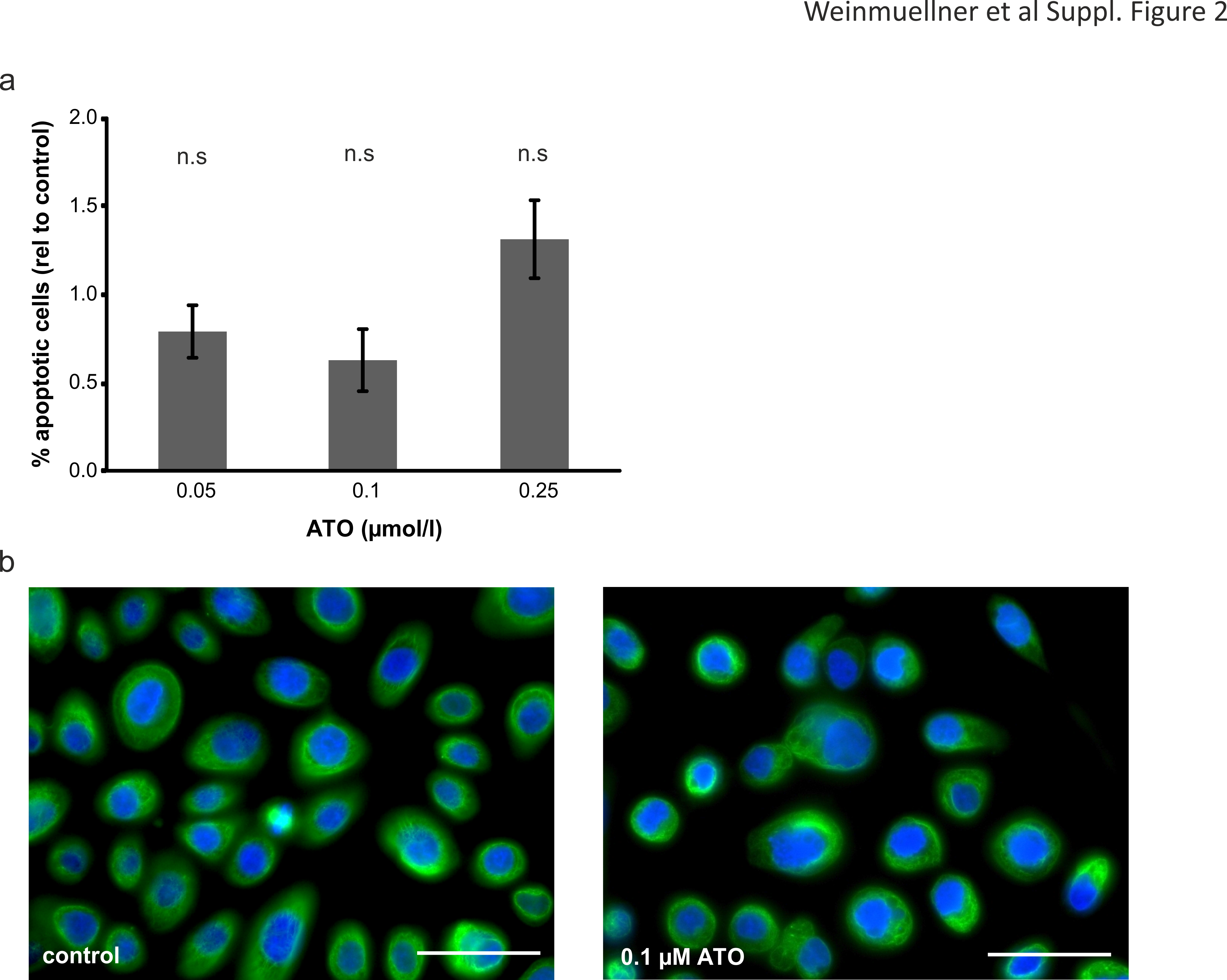

Supplement: Supplementary file 2 — Chronic arsenic treatment has no effect on apoptosis levels in NHEK/SVTERT3-5 cells. (a) The impact of ATO treatment on chronically ATO-exposed NHEK/SVTERT3-5 cells at the indicated concentrations was investigated by AnnexinV/PI staining and subsequent FACS analysis. Percentage of cells with apoptotic features was determined after the indicated ATO treatment (corresponding to their chronic selection pressure) for 72 h. (b) 2D cell layers of chronically ATO-exposed cells were formalin-fixed and immunhistologically stained with tubulin tracker. Scale bar, 50 µM (TIFF 38047 kb) [file 204_2017_2034_MOESM2_ESM.tif]

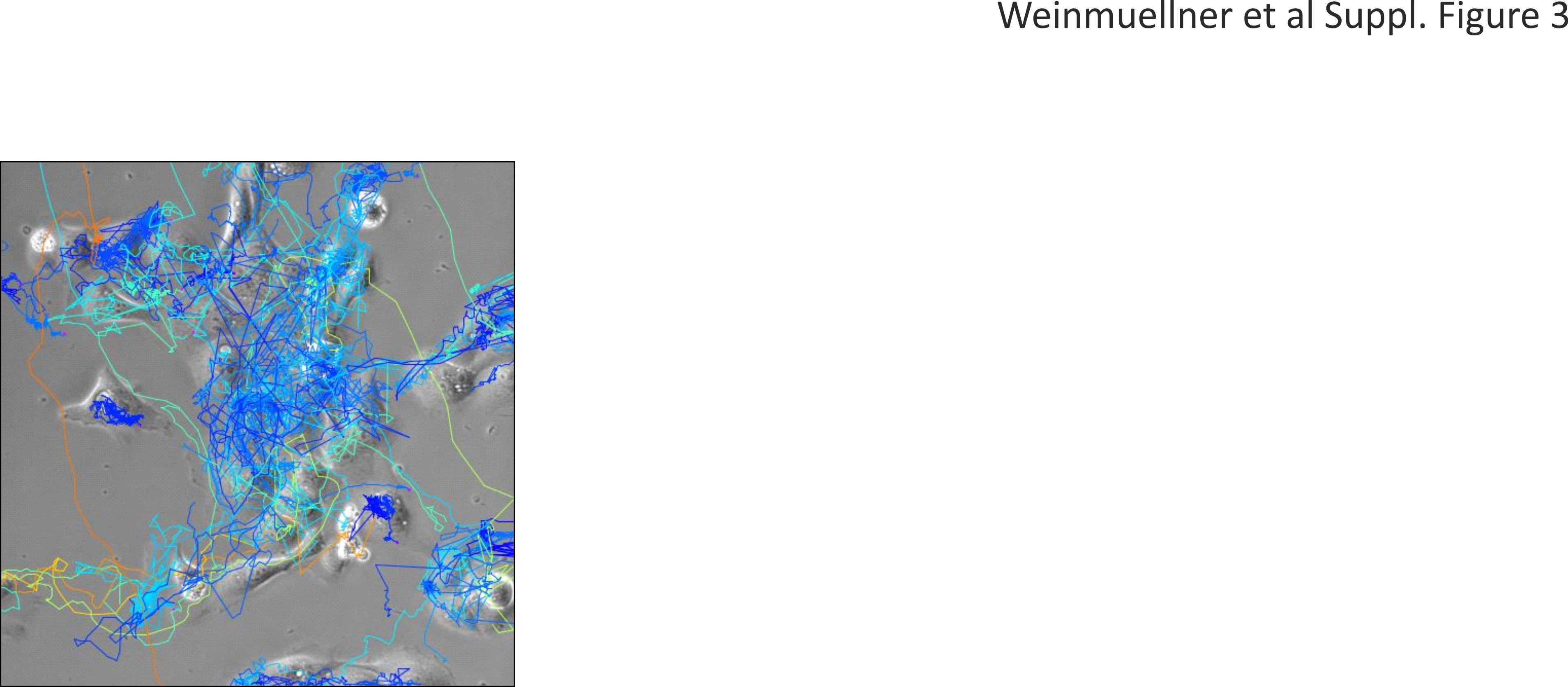

Supplement: Supplementary file 3 — Representative example of single cell migration trajectories. Migration trajectories were generated with Fiji/ImageJ using the TrackMate plug-in and Simple LAP tracker from time-lapse microscopy images. (TIFF 18701 kb) [file 204_2017_2034_MOESM3_ESM.tif]
