# Supplementary material for: Long-term exposure of immortalized keratinocytes to arsenic induces EMT, impairs differentiation in organotypic skin models and mimics aspects of human skin derangements
Source: Arch Toxicol. 2017 Aug 3;92(1):181–94. doi: 10.1007/s00204-017-2034-6 (PMC5773649; doi:10.1007/s00204-017-2034-6)
Supplement: Supplementary file 4 — Supplementary material 4 (DOCX 12 kb) [file 204_2017_2034_MOESM4_ESM.docx]

**Supplementary Table 1. Antibodies used for immunofluorescence and IHC**

| **Detected protein** | **Species** | **Dilution** | **Purchased from:** |
| --- | --- | --- | --- |
| Ki67 | mouse | 1:100 | DAKO M7240 |
| Filaggrin | mouse | 1:100 | Leica, NLC-FIL |
| Cytokeratin 10 | rabbit | 1:1000 | Biolegends, 905401 |
| Cytokeratin 14 | mouse | 1:100 | Abcam, ab7800 |
| E-Cadherin | goat | 1:100 | R&D Systems, AF648-SP |
| Alexa Fluor 488 anti mouse IgG (H+L) | donkey | 1:500 | Jackson Immunoresearch,  715-545-150 |
