# Supplementary material for: Long-term exposure of immortalized keratinocytes to arsenic induces EMT, impairs differentiation in organotypic skin models and mimics aspects of human skin derangements
Source: Arch Toxicol. 2017 Aug 3;92(1):181–94. doi: 10.1007/s00204-017-2034-6 (PMC5773649; doi:10.1007/s00204-017-2034-6)
Supplement: Supplementary file 5 — Supplementary material 5 (DOCX 12 kb) [file 204_2017_2034_MOESM5_ESM.docx]

**Supplementary Table 2. Primary Antibodies used for Westernblot**

| **Detected protein** | **Species** | **Dilution** | **Purchased from:** |
| --- | --- | --- | --- |
| E-Cadherin | rabbit | 1:1000 | Cell Signalling Technology |
| TCF8/ZEB1 | rabbit | 1:1000 | Cell Signalling Technology |
| Slug | rabbit | 1:1000 | Cell Signalling Technology |
| β-Catenin | rabbit | 1:1000 | Cell Signalling Technology |
